# Supplementary material for: Hierarchically Porous Carbon Nanosheets from One-Step Carbonization of Zinc Gluconate for High-Performance Supercapacitors
Source: Int J Mol Sci. 2023 Sep 15;24(18):14156. doi: 10.3390/ijms241814156 (PMC10531767; doi:10.3390/ijms241814156)
Supplement: Supplementary file 1 [file ijms-24-14156-s001.zip › ijms-2590259-supplementary.pdf]

## Supporting Information

Hierarchically Porous Carbon Nanosheets from One-step Carbonization of Zinc Gluconate for High-Performance Supercapacitors

Zhiwei Tian [1,†], Zhangzhao Weng [2,†,*], Junlei Xiao [1], Feng Wang [1], Chunmei Zhang [3] and Shaohua Jiang [1,*]

The kinetics of the charge storage process can be evaluated ty the CV curves, where the total charge storage can be separated as the capacitive effects and diffusion-controlled charge storage, which can be expressed by the following eq (S1) and eq (S2), where the i is the peak current, v is the scan rate, a and b are the fitting parameters:

$$i_{total} = i_{cap} + i_{diff} = av^b \tag{S1}$$

$$\log i = \log a + \mathrm{b}\log v \tag{S2}$$

The capacitance contributions from the surface capacitive effects and the diffusion-controlled intercalation process can be quantitatively distinguished by eq (S3) and eq (S4). Where $k_1v$ and $k_2v^{1/2}$ are the capacitive effects and diffusion-controlled intercalation process contributions, respectively:

$$I(V) = k_1v + k_2v^{1/2} \tag{S3}$$

$$i(V)/v^{1/2} = k_1v^{1/2} + k_2 \tag{S4}$$

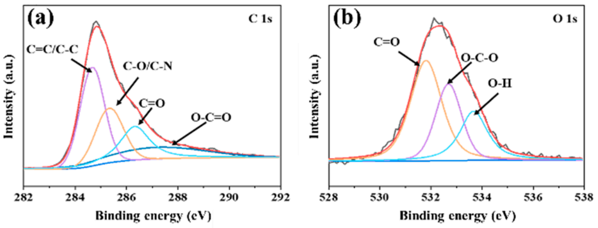


**Figure S1** High-resolution of C 1s(a) and O 1s(b) of ZnPCN-1.

**Table S1** Textural parameters of all samples.

| Samples | $S_{BET}$ (m$^2$ g$^{-1}$) [a] | $V_t$ (cm$^3$ g$^{-1}$) [b] | $D_P$ (nm) [c] | N content (at %) |
|---|---|---|---|---|
| ZnPCN-0.5 | 1135 | 0.47 | 3.32 | 2.95 |
| ZnPCN-1 | 1162 | 0.49 | 3.33 | 4.57 |
| ZnPCN-2 | 1146 | 0.48 | 3.38 | 4.40 |

[a] BET surface area.

[b] Total pore volume.

[c] Desorption average pore diameter by BET method.

**Table S2** Comparison of ZnPCN-1 with the reported work.

| Samples | Current density | Specific capacitance | Electrolyte | Ref. |
|---|---|---|---|---|
| ZnPCN-1 | 1 A g$^{-1}$ | 221 F g$^{-1}$ | 6 M KOH | This work |
| C9-250k-12 | 0.5 A g$^{-1}$ | 197 F g$^{-1}$ | 1 M $Na_2SO_4$ | [1] |
| MA6 | 0.5 A g$^{-1}$ | 182 F g$^{-1}$ | 3 M KOH | [2] |
| SAK | 2 mVs$^{-1}$ | 129 F g$^{-1}$ | PVA/LiCl | [3] |
| SSP-900 | 1 A g$^{-1}$ | 199 F g$^{-1}$ | 3 M KOH | [4] |
| Gna-CA | 1 A g$^{-1}$ | 140 F g$^{-1}$ | 1 M $Na_2SO_4$ | [5] |

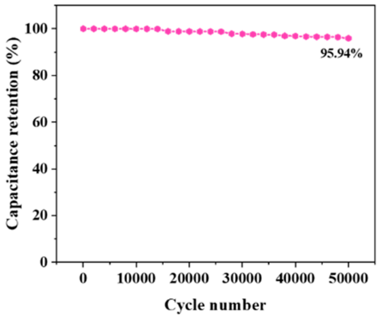


**Figure S2** Cycling performance of ZnPCN-1 at a current density of 50 A g$^{-1}$ in 6 M KOH.

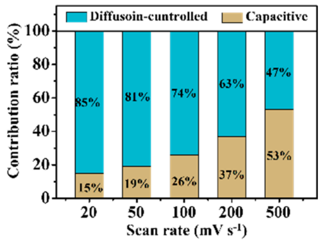


**Figure S3** Capacitance contribution ratios of ZnPCN-1 at different sweep rates.

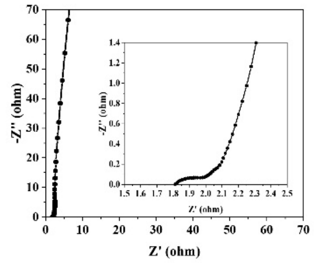


**Figure S4** EIS plots of ZnPCN-1//ZnPCN-1 in 6 M KOH.

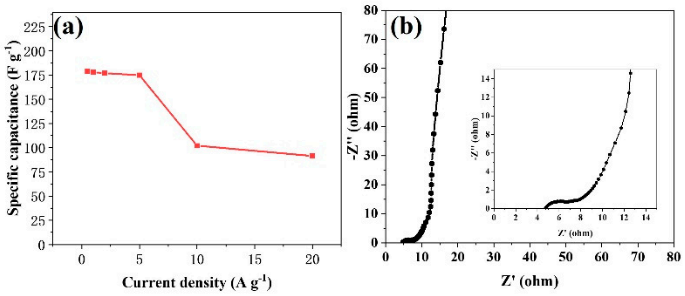


**Figure S5** Rate performance (a), EIS plots (b) of ZnPCN-1//ZnPCN-1 in $Et_4NBF_4$.

**Table S3** Comparison of ZnPCN-1//ZnPCN-1 with the reported work.

| Samples | Energy density (Wh $Kg^{-1}$) | Power density (W $Kg^{-1}$) | Electrolyte | Ref. |
|---|---|---|---|---|
| ZnPCN-1//ZnPCN-1 | 17.2 | 499 | 6 M KOH | This work |
| ZnPCN-1//ZnPCN-1 | 153.4 | 1242 | 1 M $Et_4NBF_4$ | This work |
| WC-E-100-48//WC-E-100-48 | 11.0 | 26.3 | 6 M KOH | [6] |
| WBMs-800//WBMs-800 | 9.4 | 227 | 6 M KOH | [7] |
| $Co(OH)_2$@CW//CW | 6.5 | 236 | 2 M KOH | [8] |
| NiCo-P//CW | 12.1 | 395 | 2 M KOH | [9] |
| N-M-O//Carbon | 20.1 | 226 | 1 M $Na_2SO_4$ | [10] |
| $MnO_x$/PANI//Carbon | 37.0 | 800 | $H_2SO_4$/PVA | [11] |

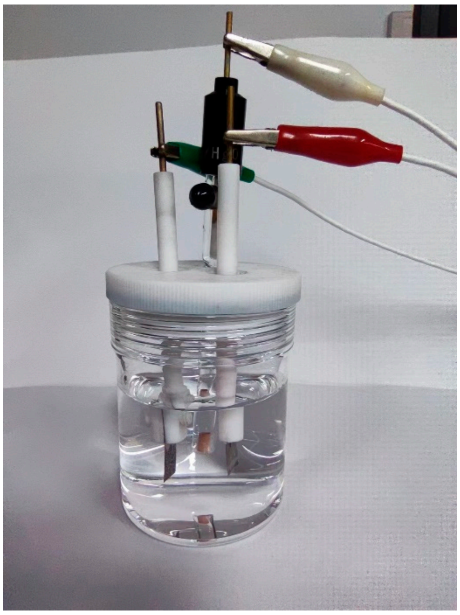

**Figure S6** Photograph of the three-electrode system.

References


1. Zhang, W.; Li, W.; Li, S., Self-template activated carbons for aqueous supercapacitors. *Sustainable Materials and Technologies* **2023,** 36, e00582.
2. Khajonrit, J.; Sichumsaeng, T.; Kalawa, O.; Chaisit, S.; Chinnakorn, A.; Chanlek, N.; Maensiri, S., Mangosteen peel-derived activated carbon for supercapacitors. *Progress in Natural Science: Materials International* **2022,** 32, (5), 570-578.
3. Zhang, X.; Zhao, M.; Chen, Z.; Yan, T.; Li, J.; Ma, Y.; Ma, L., The application of biomass-based carbon materials in flexible all-solid supercapacitors. *Journal of Materials Science: Materials in Electronics* **2022,** 33, (19), 15422-15432.
4. Pandey, K.; Jeong, H. K., Coffee waste-derived porous carbon based flexible supercapacitors. *Chemical Physics Letters* **2022,** 809, 140173.
5. Fuertes, A. B.; Sevilla, M., Hierarchical Microporous/Mesoporous Carbon Nanosheets for High-Performance Supercapacitors. *ACS Applied Materials & Interfaces* **2015,** 7, (7), 4344-4353.
6. Wang, F.; Cheong, J. Y.; Lee, J.; Ahn, J.; Duan, G.; Chen, H.; Zhang, Q.; Kim, I. D.; Jiang, S., Pyrolysis of Enzymolysis-Treated Wood: Hierarchically Assembled Porous Carbon Electrode for Advanced Energy Storage Devices. *Advanced Functional Materials* **2021,** 31, (31), 2101077.
7. Ma, Y.; Yao, D.; Liang, H.; Yin, J.; Xia, Y.; Zuo, K.; Zeng, Y.-P., Ultra-thick wood biochar monoliths with hierarchically porous structure from cotton rose for electrochemical capacitor electrodes. *Electrochimica Acta* **2020,** 352, 136452.
8. Wang, Y.; Lin, X.; Liu, T.; Chen, H.; Chen, S.; Jiang, Z.; Liu, J.; Huang, J.; Liu, M., Wood-Derived Hierarchically Porous Electrodes for High-Performance All-Solid-State Supercapacitors. *Advanced Functional Materials* **2018,** 28, (52), 1806207.
9. Chen, Y.; Hou, H.; Liu, B.; Li, M.; Chen, L.; Chen, C.; Wang, S.; Li, Y.; Min, D., Wood-derived scaffolds decorating with nickel cobalt phosphate nanosheets and carbon nanotubes used as monolithic electrodes for assembling high-performance asymmetric supercapacitor. *Chemical Engineering Journal* **2023,** 454, 140453.
10. Xuan, H.; Xu, Y.; Liang, T.; Liang, X.; Xie, Z.; Han, P.; Du, Y., Molten Salt Synthesis of Na-Mn-O Composites as Electrode Materials for High-Performance Supercapacitors. *ChemElectroChem* **2019,** 6, (6), 1838-1845.
11. Ye, Q.; Luo, Y.; Cen, Q.; Dong, R.; Luo, T.; Xu, X.; Wang, F.; Li, B., In situ hybridization of polyaniline on Mn oxide for high-performance supercapacitor. *Journal of Energy Storage* **2021,** 36, 102330.
